# Microtiming Deviations and Swing Feel in Jazz

**DOI:** 10.1038/s41598-019-55981-3

**Published:** 2019-12-27

**Authors:** George Datseris, Annika Ziereis, Thorsten Albrecht, York Hagmayer, Viola Priesemann, Theo Geisel

**Affiliations:** 1 Max Planck Institute for Dynamics and Self-Organization, 37077 Göttingen, Germany; 2 Department of Physics, Georg-August-University Göttingen, 37073 Göttingen, Germany; 3 Georg-Elias-Mueller Institute for Psychology, Georg-August-University Göttingen, 37073 Göttingen, Germany; 4 Bernstein Center for Computational Neuroscience, 37077 Göttingen, Germany

**Keywords:** Perception, Emotion, Social behaviour, Human behaviour, Statistics

## Abstract

Jazz music that swings has the fascinating power to elicit a pleasant sensation of flow in listeners and the desire to synchronize body movements with the music. Whether microtiming deviations (MTDs), i.e. small timing deviations below the bar or phrase level, enhance the swing feel is highly debated in the current literature. Studies on other groove related genres did not find evidence for a positive impact of MTDs. The present study addresses jazz music and swing in particular, as there is some evidence that microtiming patterns are genre-specific. We recorded twelve piano jazz standards played by a professional pianist and manipulated the natural MTDs of the recordings in systematic ways by quantizing, expanding and inverting them. MTDs were defined with respect to a grid determined by the average swing ratio. The original and manipulated versions were presented in an online survey and evaluated by 160 listeners with various musical skill levels and backgrounds. Across pieces the quantized versions (without MTDs) were rated slightly higher and versions with expanded MTDs were rated lower with regard to swing than the original recordings. Unexpectedly, inversion had no impact on swing ratings except for two pieces. Our results suggest that naturally fluctuating MTDs are not an essential factor for the swing feel.

## Introduction

The swing feel is a major feature in most jazz music performances, but what is it that actually makes a piece of music swing? For many years, musicologists have attempted to describe and explain this phenomenon. Already in 1937, the rhythmic characteristic of swing was described as “an almost imperceptibly hurried accent of the second and fourth beats” and further, “the word really defies definition, for most musicians protest that swing cannot be regarded wholly as a matter of rhythm, but that it depends rather on a personal emotional response to rhythm that cannot be written in any musical notation^1^”.

Today, the swing feel is sometimes classified as a kind of groove in jazz music^2,3^. Swing and groove are not generally interchangeable (there is no swing without groove, but there may be groove without swing) but both concepts are related. Both swing and groove are expected to induce a pleasant sensation of wanting to move along with the music. These two aspects (enjoyment and entrainment) are commonly used to define groove (see e.g.^4^).

There have been some rhythmic characteristics of a piece identified, like syncopation^5,6^ (i.e. rhythmic stresses on the offbeat) or the swing ratio^7–10^, to influence groove. Specifically in jazz the *swing ratio*, i.e. the length ratio of consecutive 8^th^ notes in the typical long-short pattern^7^, has been studied extensively^8^. The variation of the swing ratio within a piece and between musicians has been related to other musical variables like intervals, articulation, harmony and melody^9^. It is also conjectured to emphasize important structural aspects of the music, to draw attention to a soloist’s phrase^10^, and to facilitate the perception in multiple onsets of notes by contrasting timbres.

The influence of the swing ratio, along with other minute timing deviations known as *microtiming deviations* (MTDs), on the swing feel and groove has generated considerable research interest^8,11–25^.

A consistent finding is that, although professional musicians can play extremely accurate, they will not match perfectly a hypothetical systematic grid produced by a metronome but show small timing fluctuations. Many musicians argue that MTDs are important for the swing feel as reflected in Charles Keil’s theory of *participatory discrepancies* (PD-Theory^26^). According to this theory it “is the little discrepancies within a jazz drummer’s beat, between bass and drums, between rhythm section and soloists, that create ‘swing’ and invite us to participate”^26^. From this perspective, MTDs create tension^3,27^, liven up the music and elicit swing, rather than reflecting inaccuracies.

On the other hand, empirical studies performed on funk, samba, jazz or rock music have failed to find a positive impact of MTDs on groove^11–15^. Music without MTDs was rated more “groovy” than music with MTDs^14,15^ and increasing the size of MTDs consistently led to lower groove ratings^15–19^. In only two studies, patterns with very small MTDs resulted in higher groove ratings than no MTDs^16,18^. Not only the magnitude but the pattern of MTDs was shown to be impactful^14,17,23^. Attempts to explain the relation between timing deviations and groove refer to predictive timing^28^ (i.e. how predictable a rhythm is), the complexity of the music structure^29–31^, and syncopation^5,6,32^, all of which relate to the detectability of the rhythmical structure and synchronization to a beat. It remains unclear, however, whether these results directly apply to MTDs in swinging music. There is some evidence that both the listeners’ familiarity and expectation of microtiming patterns as well as the MTDs patterns vary across genres^14–16,23,33^. Jazz music, compared to other groove related music, could therefore elicit different expectations with regard to (micro-)timing patterns.

Besides in empirical and qualitative studies, musical MTDs have been studied quantitatively by analyzing naturally occurring MTDs^33–36^. Specifically in^34^ it was shown that the sequence of MTDs produced from humans following a metronome tick is not white noise, but instead power-law correlated. This finding applies not only to simple tapping-like time series, but also to real music recordings^33,36^ and musicians’ interactions^35^. Interestingly, the variability and magnitude of MTDs in jazz music was found to be larger than in rock^33^. This motivates analyzing jazz music, rather than e.g. rock, since the MTDs are more likely to be above the auditory perception threshold due to their bigger magnitude^37^.

## Aim of the Study

**Figure 1:**
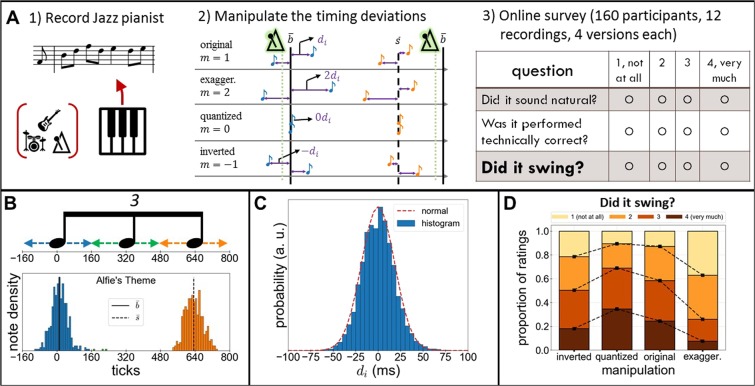
(**A**) Experimental setup. (1) A professional pianist was recorded performing jazz standards, while listening to quantized bass and drum tracks. (2) We determined the average base note position $\bar{b}$ and swing note position $\bar{s}$ for each recording. The microtiming deviations ${d}_{i}$ of the notes were defined with respect to $\bar{b},\bar{s}$ and were subsequently manipulated using three different manipulations: exaggerated, quantized and inverted. (3) Original and manipulated recordings were then used in an online survey where musicians judged them. The audio samples used are available online^38^. (**B**) (top) Sketch of the range of ticks that an 8^th^ note triplet covers. (bottom) Note position density (modulo the quarter note), and mean note positions $\bar{b},\bar{s}$. Note classification: blue = base ($b$), orange = swing ($s$), green = disregarded. 1 tick is 1/960-th of a quarter note (dimensionless unit of time). (**C**) Histogram of micro-timing deviations, measured in milliseconds, across all pieces and both note types $b,s$. Plotted is also a normal distribution with the same mean (0.08 ms) and variance (18.39 ms), showing the excellent fit. (**D**) Proportion of answers for “Does it swing?”, combining all pieces and participants (dashed lines are guide to the eye reflecting cumulative proportions of answers). The main result of the survey, that the quantized version is preferred, is evident.

As outlined above, the role of MTDs for groove for the swing feel has remained quite controversial. In the present paper we address the question, whether naturally occurring MTDs of a soloist do elicit a stronger swing feel compared to other MTDs patterns. This is done first by recording a professional pianist and manipulating the MTDs of the recordings (see Section 1.2) and second by carrying out an online survey for which we test the manipulated and original performances. In line with the (widespread) opinion that musicians “can feel it, but you just can’t explain it”^38^, p.8-10 we let 160 listeners rate the swing feel in different versions of manipulated and original recordings. We aimed for a diverse sample of jazz and non-jazz, expert and non-expert listeners (see 2.2.1) to control for possible genre- and skill-dependent moderation effects. Furthermore, we chose to record and manipulate a broad variety of twelve jazz pieces (see Table 1), differing in several aspects like swing ratio, tempo and syncopation. This ensures that our findings generalize across different pieces and do not depend on specific aspects of a piece. Besides quantizing, i.e. eliminating the MTDs, and augmenting them by a factor of 2, we also inverted them (by multiplying by a factor of -1) thereby changing the respective microtiming pattern without changing its magnitude. Inverting means that playing ahead of the beat (“dragging”) becomes playing before the beat (“rushing”), and vice-versa. The process we just described is also visualized in Fig. 1A. While the rating of the swing feel was our main focus of interest, we additionally implemented two control questions about whether the piece sounded natural and was played technically correctly.

**Table 1:** Properties of micro-timing deviations (MTDs) of recordings. Notice that mean note positions $\bar{b},\bar{s}$ are given in ticks (MIDI standard), but the standard deviations of the MTDs ${\sigma }_{b},{\sigma }_{s}$ are in milliseconds (see Supporting Information for more details on the units). $r$ and ${\delta }_{r}$ are the average swing ratio as defined in Eq. (1) and its standard deviation, respectively (see 5.1). ${\rho }_{\alpha \to \beta }$ is Spearman’s rank correlation coefficient between microtiming deviations of pairs of notes of types $\alpha ,\beta$ (see 5.2). All recording versions used are available as Supporting Information. *Title of piece “Don’t Get Around Much Anymore” was shortened to fit.

Recording	BPM	$\bar{{\boldsymbol{b}}}$	$\bar{{\boldsymbol{s}}}$	${{\boldsymbol{\sigma }}}_{{\boldsymbol{b}}}$ (ms)	${{\boldsymbol{\sigma }}}_{{\boldsymbol{s}}}$ (ms)	${\boldsymbol{r}}$	${{\boldsymbol{\delta }}}_{{\boldsymbol{r}}}$	${{\boldsymbol{\rho }}}_{{\boldsymbol{b}}\to {\boldsymbol{s}}}$	${{\boldsymbol{\rho }}}_{{\boldsymbol{s}}\to {\boldsymbol{b}}}$
Alfie’s Theme	135	11	634	16.94	18.3	1.99	0.39	0.22	0.51
Blue Monk	140	0	627	18.85	17.5	1.93	0.39	0.43	0.57
Don’t Get Around*	140	24	644	20.44	19.18	2.11	0.51	0.48	0.51
Doxy	130	1	624	15.31	17.85	1.89	0.33	0.21	0.59
Four	170	3	602	17.7	17.01	1.73	0.38	0.67	0.75
In a Mellow Tone	160	−7	623	20.1	21.79	1.94	0.58	0.72	0.69
Jordu	150	−1	603	19.11	17.31	1.73	0.33	0.39	0.66
Now’s The Time	190	−20	582	15.55	21.01	1.62	0.45	0.53	0.73
Paper Moon	135	−2	635	18.73	17.34	2.0	0.41	0.41	0.46
Serenade to a Cuckoo	140	22	634	21.6	20.15	2.0	0.42	0.1	0.59
So What	160	4	582	18.48	15.59	1.57	0.32	0.67	0.8
Yardbird Suite	180	7	596	21.12	20.38	1.71	0.51	0.59	0.53

### Hypotheses

According to PD-theory, the original versions of the pieces should be rated as the most swinging, having the optimal MTDs pattern. In contrast, empirical research suggests that the quantized versions elicit the strongest groove due to higher predictability of the pulse. From both perspectives we expect a reduced swing feel for versions with exaggerated MTDs. Furthermore, if there was an objectively “appropriate” use of MTDs to create swing, inversion should lead to lower swing ratings as the pianist’s specific style of playing would be erased.

## Defining and Manipulating the Microtiming Deviations

The pianist was recorded effectively with a metronome, since during the recording he was listening to a backing track consisting of quantized (but human-played) bass and drums tracks. The tracks were quantized on ${8}^{{\rm{th}}}$ note triplets (see Supporting Information for more details of the recording process). As the recorded pieces were in a jazz style, the phrasing of notes is based on ${8}^{{\rm{th}}}$ note triplets, out of which only the first and third notes are played (“swung ${8}^{{\rm{th}}}$ notes”). This is also confirmed by the note density of a recording shown in Fig. 1B. As the swing ratio is variable and not always the “typical” value of 2:1 (i.e. perfect triplets, see also Table 1), there is an ambiguity in defining the MTDs. We found the following definition based on an average swing ratio to be most appropriate for this study.

Let us first consider the first notes of all triplets, which we call “base” notes $b$. These notes are supposed to follow the metronome click, but of course are not played exactly on it and instead deviate (a sketch is also shown in Fig. 1). However, it may be that the pianist is intentionally playing behind or ahead of the click, and we would want to keep this aspect of the performance intact. What we did was calculate the average base note position $\bar{b}$ with respect to the metronome click individually for each recording. Then, the MTDs of the base notes ${d}_{i}$ is simply their deviation from their mean position $\bar{b}$ (modulo the quarter note). This is illustrated in Fig. 1A (middle panel, top row, blue notes) and **B** (blue notes, first part of triplet). The same process was applied to the third notes of all triplets, which we call “swing” notes $s$. For them, the MTDs are measured with respect to $\bar{s}$, see Fig. 1 (orange colors). An advantage of defining the MTDs this way is that it respects the *average* swing ratio of the recording (see results section). A more detailed definition of the MTDs, based on the internal format of MIDI data, is presented in the Supporting Information.

After establishing the MTDs, we manipulated them by multiplying each deviation ${d}_{i}$ with a constant factor $m$. In this study we chose $m=0,2,\,-\,1$. This process is shown in Fig. 1A middle panel.

Notes that fell in between the first and third part of an ${8}^{{\rm{th}}}$ note triplet were not manipulated. However, they represented on average only 0.7% of the total notes and at most 1.8% (Serenade to a Cuckoo). Using a manipulation of $m=0$ closely corresponds to the so-called “quantization” procedure, a well-known process in music production. Our “quantization”, however, keeps intentional playing consistently ahead or behind the beat intact (if there is any). Lastly, we point out that no other aspect of the performance was changed. The pianist’s choice of what chords to play, what phrasing to use, how to anticipate notes, etc. was left untouched. In addition, note intensity (dynamic level, e.g. piano or forte), duration and even sustain pedal usage were left intact.

## Results

### Statistical properties of the recordings

First, we investigated the basic statistical properties of MTDs (Table 1). Across pieces, the MTDs were approximately following a normal distribution (Fig. 1C shows aggregate behavior, but individual pieces also follow a normal distribution - not shown) with $\sigma \approx 18.5\pm 2$ ms (for either $b,s$, see Table 1). Similar observations were made in previous studies^33,34^. The fairly invariant magnitude (as seen in the columns of ${\sigma }_{b}$ and ${\sigma }_{s}$ of Table 1) of the MTDs is noteworthy, because the individual pieces differ in tempo, complexity and many other aspects. This suggests that the magnitude of these MTDs is probably not controlled by the performer, but reflects instead a “human error” from a temporally exact performance. In Fig. 1 we show the computed distribution of MTDs of all played notes (all recordings and both $b,s$, in order to increase the amount of data for a better fit). The result is compared with a normal distribution with same mean and std.

The average swing ratio $r$, defined in detail in section 5.1, characterizes the average position of a swing note $s$ with respect to the metronome click (see sec. 5.1). In jazz music $r$ typically varies between 1:1 (straight 8${}^{{\rm{th}}}$s) to 3:1 (dotted-8${}^{{\rm{th}}}$ and 16${}^{{\rm{th}}}$ notes in alternation), depending on tempo and musician^7^. As expected, the swing ratio decreased as the tempo of the recordings increased (Table 1). In our recordings, the maximum $| \bar{b}|$ is approximately 10 ms (24 ticks), which puts it at the limits of auditory thresholds. This means that playing “behind” or “ahead of” the beat was not pronounced in our recordings.

### Online survey

After the initial assessment of the musical background, the participants were presented with the core part of the survey, which is displayed in Fig. 1A (panel 3). For 12 audio samples, each lasting 60 seconds, the participants answered a questionnaire identical with that of the figure. The audio samples used in the survey are available online^39^.

In this online survey we applied a mixed within-between-subjects design in which only one version for each piece was presented to a participant. We thus obtained a global assessment of the swing feel of the individual pieces and avoided prompting our participants (to try) to detect differences between manipulations and compare them.

#### Participants

In the online survey we had 160 participants (42 females, 115 males, 3 NA, *M*${}_{age}$ = 39.6 $\pm$ 15.5 years), who rated at least six out of the twelve pieces. Of those participants, 24.4% were professional jazz, 15% semi-professional jazz, 29.4% amateur jazz and 26.3% non-jazz musicians as well as 5% jazz-loving non-musicians. Non-musicians without a penchant for jazz were excluded from the survey. A more detailed description of the procedure of the listening study, the recruitment and assessment of the participants’ musical background is presented in the Supporting Information.

#### Swing ratings

**Figure 2:**
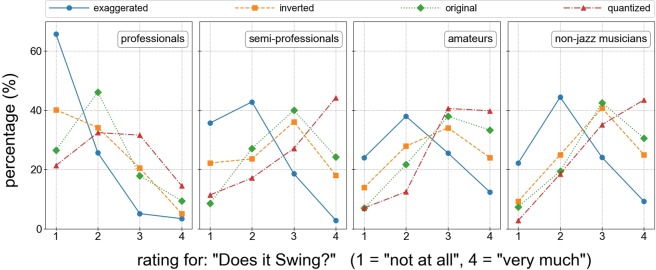
Percentages of ratings for the question *“Does it swing?”* for the different manipulations, collapsed across pieces. Each subplot shows the percentages of the ratings for a specific group of musical expertise, while all subplots use the same legend. Note that percentages (points) of one condition (one colour) add up to 100% accumulated across response categories. The figure shows that the quantized version (red) is on average preferred over all others, the exaggerated version (blue) fares worse than all others and that original and inverted versions (green and orange) have very similar ratings.

The results of the online survey indicate that the manipulation of MTDs had an impact on swing ratings. Fig. 2 displays the proportions of the participants’ ratings on each point of the swing scale (1 = not at all, to 4 = very much) shown separately for the musicians’ groups. Across all musicians’ groups the exaggerated versions elicited the lowest swing ratings, as the peak of the distributions is shifted to the lower end of the swing rating scale. The opposite was found for the quantized versions, for which the majority of participants gave the highest swing ratings. The distribution for the inverted version is similar to the original. Both were rated as more swinging compared to the exaggerated but not as much as the quantized versions. Additionally to the manipulation, a difference between the musicians’ groups was found. Professional jazz musicians gave overall lower swing ratings, independent of the version of a piece. This can be seen in Fig. 2, where the distributions for professionals in the lower ratings is shifted further to the left in contrast to the other groups.

The inferential statistical results were obtained with a proportional odds mixed model that was fitted to the data (see Section 5.4). As reference categories the original version and the group of non-jazz musicians were chosen. Table 2 shows the estimated model parameters for the swing ratings.

**Table 2:** Proportional odds mixed model for swing fitted with Laplace Approximation. The estimates display the effects of the manipulations and musicians’ categories. As references, the original version of a piece and the group non-jazz musicians were used.

	${\boldsymbol{\beta }}$	*SE*	*p*
**Condition**			
exaggerated	−1.490	0.242	$<$0.001
inverted	-0.287	0.236	0.224
quantized	0.503	0.241	0.037
Musicians’ Category			
amateur jazz	0.043	0.325	0.895
professional jazz	−1.815	0.341	$<$0.001
semiprof. jazz	-0.449	0.393	0.254
**Condition x**			
Musicians’ Category			
exagg. x amateur jazz	−0.092	0.331	0.781
invert. x amateur jazz	−0.138	0.328	0.674
quant. x amateur jazz	−0.008	0.335	0.982
exagg. x prof. jazz	−0.642	0.368	0.082
invert. x prof. jazz	−0.356	0.345	0.302
quant. x prof. jazz	0.100	0.345	0.775
exagg. x semiprof. jazz	−0.354	0.404	0.380
invert. x semiprof. jazz	−0.036	0.399	0.929
quant. x semiprof. jazz	0.269	0.409	0.510
Threshold coefficients	**Estimate**	***SE***	
1$$| $$2	−2.961	0.300	
2$$| $$3	−0.934	0.291	
3$$| $$4	1.027	0.291	

Quantization and exaggeration of the MTDs led to significantly different swing ratings compared to the original recordings. The Odds Ratio (OR) for the quantized (compared to the original) version to be swinging were 1.65 (95% CI = 1.03–2.65, $p=0.037$) and for the exaggerated versions 0.23 (95% CI = 0.14 –0.36, $p < 0.001$), respectively. Hence, by averaging and removing the variability of MTDs, higher swing ratings were found, whereas by increasing the magnitude of MTDs the swing feel was reduced. More precisely, compared to the original version, the quantized version of a piece was 1.65 times more likely to get higher swing ratings, whereas swing ratings of an exaggerated version of a piece were 4.42 times more likely to be lower than of an original version, keeping the musicians’ category constant. Averaging across all pieces, inversion of MTDs did not significantly lower swing ratings (*OR* = 0.75, 95% CI = 0.47 - 1.19, $p=0.224$). Indeed, swing ratings of the inverted versions were similar to those of the original recordings for the majority of pieces. Only for two out of twelve pieces (Jordu and Yardbird Suite), inversion had a negative impact on swing ratings.

**Figure 3:**
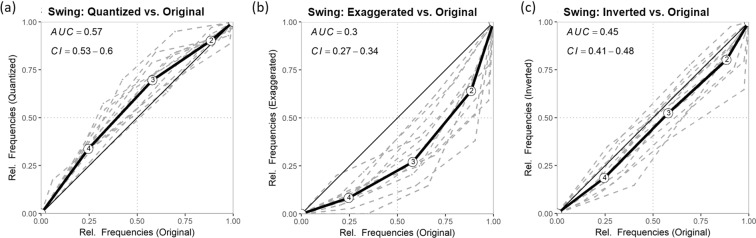
Receiver Operating Characteristic (ROC) curves for swing ratings across pieces. The discriminability between original versions and the respective manipulation are indicated by the area under the curve (AUC). The curves display cumulative proportions for each answer category, starting from 4 (very much) to 1 (not at all), of which the manipulation is plotted on the original version. The solid line represents the average across pieces. Dashed lines are ROC of the individual pieces, marking the variability between pieces. AUC below 0.5 indicate a preference for the original version over the manipulation. Although there is a variability between pieces in the strength of the effect of the manipulation, the direction of the effects is mainly consistent within manipulations (e.g. exaggerated have lower swing ratings compared to the original versions across all pieces).

Receiver Operating Characteristics (ROC) analyses were performed for each piece and across all pieces to test the discriminability of conditions overall and at the piece level. For visualization, the relative frequencies on every rating point of one condition, e.g. original, were plotted against the relative frequencies of another condition, e.g. the inverted version of a piece, resulting in a more or less curved line. Deviation from the diagonal indicate that participants differentiate between conditions. The area under the curve (AUC) was computed and confidence intervals were estimated using DeLong tests^40^. If confidence intervals include 0.5 (which would reflect visually the straight diagonal) no statistically significant difference between conditions are indicated. Figure 3 shows ROCs across all pieces. ROCs for the individual pieces can be obtained from the Supporting Information. The effect of inversion on Jordu and Yardbird Suite was shown by areas under the curves (AUC) differing significantly from 0.5 (Jordu: *AUC* = 0.32, 95% CI = 0.20–0.44; Yardbird Suite: *AUC* = 0.34, 95% CI = 0.22–0.46). In these two cases, the inverted versions were rated lower in swing compared to the original version. We also performed a sanity check, searching for possible correlations of the AUC of quantized versus original with other aspects of the recordings, e.g. tempo, intensity, note density, etcâĂę to possibly identify factors that might be related to differences between the conditions. Since we found no significant correlations, we present this information in the Supporting Information.

The musical background of participants showed a main effect on swing ratings. Professional jazz musicians were observed to be generally more harsh in their judgments and gave overall lower swing ratings (*OR* = 0.16, 95% CI = 0.08–0.32, $p < 0.001$) independent of the version of a piece. As there was no significant interaction of musicians’ group and condition (all $p > 0.05$) the manipulation seems to have similar effects on the swing ratings independent of the participants’ musical background.

#### Additional measures

With regard to naturalness, only the exaggerated differed from the original versions and were perceived as less natural (*OR* = 0.27; 95% CI = 0.17–0.43, $p < 0.001$). Naturalness ratings of quantized (*OR* = 0.98; 95% CI = 0.61–1.57, $p=0.935$) and inverted (*OR* = 0.64; 95% CI = 0.40–1.02, $p=0.060$) versions were similar to the original version. Quantized and original versions were similarly rated as being played technically correct (*OR* = 1.27; 95% CI = 0.80–2.04, $p=0.351$). Exaggerated (*OR* = 0.08; 95% CI = 0.05–0.13, $p < 0.001$) and inverted (*OR* = 0.49; 95% CI = 0.31–0.79, $p=0.001$) versions were rated lower on this dimension. All model parameters can be found in the Supporting Information.

## Discussion

In the present study we tested whether the manipulation of MTDs, originating from a professional musician’s performance, affects the swing feel. To do so, we created different versions of the original recordings by changing the MTDs in a systematic way and compared the ratings of the manipulated and the original versions of the pieces. Our results show that the manipulation of MTDs has an impact on the swing feel. Contrary to the PD-theory^27^, however, naturally occurring MTDs do not seem essential for the swing feel, as the versions without MTDs elicited the highest self-reported swing feel. This is in line with other empirical studies, which suggest that a very tight and regular timing is more likely to enhance entrainment and groove^15–19^. Our own study dealt with jazz music and the phenomenon of swing in particular. We imagine, however, that our conclusions might generalize also to the phenomenon of groove in various other music styles.

Many musicians claim that music without MTDs sounds unnatural and machine-like. Our approach of quantizing the original recordings tried to conserve as much as possible the style introduced by the pianist. We did not synchronize the pianist’s track and the rhythm section to eliminate delay between the tracks. If the pianist chose to play consistently behind the beat to create a *laid back feel*, our procedure maintained this asynchrony in the manipulations (even though this was not significant in our recordings, see $\bar{b}$ in Table 1). Moreover, we conserved all other parts that one could regard as the “human touch” of the performance, like for example intensity and harmonization. The important difference between the original and quantized versions is that our quantization procedure eliminated fluctuations of the swing ratio within a piece and made it perfectly systematic and predictable from a temporal perspective. This might have facilitated the listeners’ entrainment. To find out, whether our quantized versions would be perceived as less natural than the original recordings, we introduced a question to control for this. Our data did not show a difference between original and quantized versions regarding naturalness. Moreover, the two dimensions (swing and naturalness) were highly correlated (*r* = 0.66) and should not be regarded as independent.

The present study showed that deviations larger than what was originally performed have a negative impact on swing, as the exaggerated versions with doubled MTDs obtained strongly decreased swing ratings. We know from previous research that the listeners’ perceptual abilities and sensitivity for timing differences can moderate the impact of MTDs^16^. Consequently, professional jazz musicians, who may be assumed to be more sensitive to timing differences, were the ones with the strictest judgments (similar to ref. ^15^). Not only did they give lowest swing rating of all groups to the originals, but also to the quantized versions. We did, however, not find significant differences in the effect of our manipulations between musicians’ groups. Like the other groups, professional jazz musicians rated the quantized versions as the most and the exaggerated versions as the least swinging.

Our study also showed that inverting the MTDs achieved very similar ratings as the original versions. This was unexpected but it can be explained once we consider the structure of the MTDs timeseries. Inverting the MTDs in a series of notes can have two different outcomes. One is that the *inter-note intervals* (INI) are left unchanged because ${d}_{i}$ and ${d}_{i+1}$ have the same sign, for some $i$. The second possibility is that a pair of short-long intervals becomes long-short instead, a case of having ${d}_{i}$, ${d}_{i+2}$ with the same sign but ${d}_{i+1}$ with opposing sign. For small magnitudes of ${d}_{i}$, like in our study, the first case should not lead to perceptual differences. One might, however, expect that the second case can have significant impact on the rating of the listeners, as it would change the local swing ratio. In contrast to this expectation, our per-piece analysis showed that the effect was substantial only for 2 out of 12 pieces (see Supporting Information) and in the other cases the ratings between inverted and original were very similar (still the original version was rated higher, on average).

To understand why original and inverted versions were rated similarly, we remind that the sequence of ${d}_{i}$ is typically long-range correlated and not random, as shown in previous studies^25,33,34,36^. This means that given some ${d}_{i}$, it is more probable that the subsequent ${d}_{i+1},{d}_{i+2}$ are numerically similar, with the similarity (i.e. correlation function) decaying as a power-law as the distance from $i$ increases.

In our case, to estimate correlations between subsequent ${d}_{i}$, we used Spearman’s rank correlation coefficient $\rho$ (see section 5.2 and Table 1). Overall, correlations of the MTDs are high ($\rho  \sim 0.5$), showing that it is much more probable for subsequent ${d}_{i}$ to have the same sign. Therefore, interchanging a short-long interval with a long-short only happened rarely, which explains the unexpectedly good rating of the inverted versions.

## Conclusions

In this work we studied the impact of MTDs on the listening experience. MTDs clearly exist in human playing, and are known to be power-law correlated^33,34,36^. It was highly debated, however, whether they have a positive impact on the perception of “groove”, or “swing”^3,8,11– 13,13–27^. By performing a study using a well controlled process of manipulating timing deviations (measured with respect to a grid determined by the mean swing ratio), as well as having a big sample pool, we are able to show that the MTDs as defined in this paper are not essential for the swing feel. Instead they rather might have a negative impact. We thus conclude that a rhythm should be persistent in its timing to yield a pronounced “swing feel”.

We want to point out that we studied a laboratory/studio setting, where we recorded a pianist performing on top of a pre-recorded (and quantized) rhythm section. In live performances in addition, musicians interact, synchronize, and adapt to each other. As we encouraged comments of the participants of our online survey, it was pointed out various times that these two aspects of interaction and adaptation were believed to be important (see Supporting Information). Thus it still remains to be seen, whether our results also carry over to a setting of interacting musicians. We intend to investigate this point in future research. The situation studied in the present paper, where solo instruments are recorded on top of a pre-recorded rhythm section, nevertheless is very common in the music industry.

## Methods

All raw piano MIDI files used in the survey, the online survey results (as raw data) and the survey participant’s comments on “what defines swing” are available online as Supporting Information. The audio samples used are also available online^39^. Computer code used for analyzing and manipulating the MIDI files is open source. We used the software MIDI.jl and MusicManipulations.jl which are hosted on GitHub and are MIT licensed, see^41^ for more details on the software. Supporting Information are provided online, with more details on the online survey and study design, recording and manipulating the recordings, and possible correlations of AUC with other measures.

### Definition of the average swing ratio

The average swing ratio $r$ quantifies the ratio of the length of a base note to a swing note. However, because not all swing notes are preceded *and* followed by a base note, we define each individual ratio as if base notes were played exactly on the metronome click. This is justified as the mean base note position $\bar{b}$ is close to 0 across all recordings, as can be see from Table 1. The swing ratio therefore is given approximately by Eq. (1) with ${p}_{i}$ the positions of the swing notes

1 $${r}_{i}=\frac{\left({p}_{i}\ \,{\rm{mod}}\,\ 960\right)}{960-\left({p}_{i}\ \,{\rm{mod}}\,\ 960\right)},\ i\in \,{\rm{swing\; notes}}\,$$

with ${p}_{i}$ the position of the note. $r$ is the average of all ${r}_{i}$ while ${\delta }_{r}$ is their standard deviation. In eq. (1) the note positions are measured in *ticks*, the unit of time used by the MIDI standard (for more details please see the Supporting Information).

### Correlations of microtiming deviations

For all base notes $b$ we find pairs of base-swing notes, the distance of which is not greater than one quarter note. We then compute $\rho$ using all possible deviations ${d}_{i}$ and ${d}_{i+1}$ for all $i$ that satisfy the aforementioned criterion. The same process is done for pairs of swing notes followed by a base note, which are not necessarily identical. In the case where multiple notes are played in a given temporal bin (e.g for chords played together with a melody note), we always only use the timing information of the earliest note onset in the bin.

### Materials and procedure of the online survey

The survey was conducted online and English or German could be selected as the survey language. After a general information about the study and collection of socio-demographic data, participants classified themselves into one of six musician categories. Depending on that, different questions on the musical background followed. Semi-professional and professional jazz musicians had to state their daily practice in hours and concerts played in the last twelve months. To assess the musical background of the remaining sample, two sub-scales (musical training and perceptual abilities) of the Goldsmith Musical Sophistication Index (Gold-MSI^42^; German version^43^) were used. Non-musicians that are not listeners of jazz were excluded from the study. Participants were asked to use headphones for better sound quality and to shield environmental noise. In a test trial, the volume could be adjusted to a comfortable level. Following that, participants were randomly assigned to one of the four stimulus sets (all participants rated all pieces and all conditions but only one version of a piece; see Supporting Information). After listening, participants rated a piece on the dimensions swing, naturalness and whether it was played technically correct, each on a 4-point scale from “not at all” to “very much”. After submitting the answers, returning to the previous page was disabled to prevent comparing pieces and modifying ratings. At the end of the questionnaire, a free text field was provided, where participants could describe what they believe makes a piece of music swing.

### Statistical analyses of online survey

To analyze swing ratings, ordinal regression was used because distances between points on the four point rating scale may not be equal. Two random effects in the model allow for variation in judgments between participants and for variation between pieces. The cumulative link mixed model was fitted with Laplace Approximation using the *clmm* function from the ordinal package in *R*^44^. To test whether adding predictors to the model would improve the data fit, Likelihood-ratio tests (LRT) were used. Derived from our theoretical model, we compared partial models (predictor variables: condition vs. condition and musicians’ group) and a full model (condition, musicians’ group and their interaction) starting with the null model (no predictor). While adding condition (${\chi }^{2}$ (3) = 308.44, $p < 0.001$, Nagelkerke’s pseudo R${}^{2}$ = 0.170) and the musical background of participants (${\chi }^{2}$ (3) = 62.58, $p < 0.001$, Nagelkerke’s pseudo R${}^{2}$ = 0.200) improved the model fit, the interaction condition x musicians’ category was not supported by the data (${\chi }^{2}$ (9) = 7.16, $p=0.621$, Nagelkerke’s pseudo R${}^{2}$ = 0.204). The model can be written as

2 $$\begin{array}{l}\,{\rm{\log }}\,\left(\frac{\,{\rm{P}}\,(\,{\rm{score}}\,(i,p)\le j)}{1-\,{\rm{P}}\,(\,{\rm{score}}\,(i,p)\le j)}\right)={\alpha }_{j}+{u}_{ip}-{\beta }_{1}x-{\beta }_{2}{y}_{i}-{\beta }_{3}x* {y}_{i}\end{array}$$

for $i=1,...,n$ and $j=1,...,3$, where $\,{\rm{P}}\,(\,{\rm{score}}\,(i,p)\le j)$ gives the probability that the swing rating of participant $i$ for piece $p$ is $j$ or lower. Different ${\beta }_{1}$ are estimated for the three different manipulations (exaggerating, quantizing and inverting compared to the original version). Similarly, ${\beta }_{2}$ represents the estimated effect of participant $i$-th musicians’ category. The ${\alpha }_{j}$ represents the cutpoints (or thresholds) in the model and ${u}_{ip}$ are the random effects for participants and pieces. To account for non-equidistant spaces of the the ordinal response scale (1–4), the model allowed for ordered but otherwise unstructured thresholds. Note that in ordinal regression we have different thresholds for the response categories but constant slopes due to the proportional odds assumption (see Table 2.

The assumption of proportional odds was tested in two ways: (1) testing the models without random factor as cumulative against proportional odds models by vector generalized linear models (ref VGAM) and (2) visually, by plotting the differences between predicted logits for varying levels of the predictors for every outcome category (Supporting Information). Both methods suggest excluding the group of jazz-loving non-musicians ($N=8$) from the analysis because including them results in a Hauck-Donner effect^45^ and variation in the proportional odds, which can lead to unstable estimates of the regression model.

### Ethics statement

All experimental procedures regarding our online survey adhere to the Declaration of Helsinki. An ethical assessment of our study was performed by the Ethikkommission of the Georg-Elias-Müller-Institut für Psychologie, which deemed that our study is non-invasive and adheres to the regulations of the Ethical Principles of the German Psychological Society (DGPs) and the Association of German Professional Psychologists (BDP). Thus, the need for ethical approval was waived by the Ethikkommission of the Georg-Elias-Müller-Institut für Psychologie. Furthermore, all participants were fully informed about the objectives and procedures of the study and gave informed consent prior to the experiment.

## Supplementary information

Supporting  Information 1

Supporting  Information 2
