# Supplementary material for: Microtiming Deviations and Swing Feel in Jazz
Source: Sci Rep. 2019 Dec 27;9:19824. doi: 10.1038/s41598-019-55981-3 (PMC6934603; doi:10.1038/s41598-019-55981-3)
Supplement: Supplementary file 1 — Supporting Information 1 [file 41598_2019_55981_MOESM1_ESM.pdf]

Supplemental Information: Microtiming Deviations and Swing Feel in Jazz

George Datseris[a,b,1,2], Annika Ziereis[c,1], Thorsten Albrecht[c], York Hagmayer[c], Viola Priesemann[a, b, d], and Theo Geisel[a,b, d]

[a]Max Planck Institute for Dynamics and Self-Organization, 37077 Göttingen, Germany
[b]Department of Physics, Georg-August-University Göttingen, 37073 Göttingen, Germany
[c]Georg-Elias-Mueller Institute for Psychology, Georg-August-University Göttingen, 37073 Göttingen, Germany
[d]Bernstein Center for Computational Neuroscience, 37077 Göttingen, Germany
[1]G.D. and A.Z. contributed equally to this work
[2]george.datseris@ds.mpg.de

December 4, 2019

Contents

1 Online survey **1**
1.1 Participants . . . 1
1.1.1 Recruitment . . . 1
1.1.2 Dropout . . . 2
1.1.3 Sample Characteristics . . . 2
1.2 Study Design . . . 2
1.3 Descriptive Statistics: Distribution of the Swing Ratings . . . 2
1.4 Cumulative Link Mixed Model . . . 3
1.5 Receiver Operating Characteristics (ROC) Analysis . . . 3
1.5.1 Swing . . . 3
1.5.2 All Dimensions: Swing, Naturalness and Technical Correctness . . . 5
1.6 Exploratory Model Selection to Predict Swing . . . 6
1.7 Visual inspection of proportional odds assumptions . . . 7
1.8 Participants' Comments . . . 7

2 Recording and Manipulating **7**

3 Detailed definition of Microtiming Deviations and the tick unit **8**

4 Possible correlations of the AUC with other measures **9**

1 Online survey

1.1 Participants

1.1.1 Recruitment

In order to recruit a sufficient number of expert listeners, personalized emails with invitation links to the survey were sent to musical conservatories, universities, big bands and choirs with the request to spread them. Participation was anonymous, voluntary and not remunerated.

1.1.2 Dropout

Out of 292 initial views, 256 participants classified themselves into the categories of musicians. From those, 13 reported to be non-musicians and were subsequently excluded from the survey. Most participants dropped out before starting the main part of the experiment. At least one music piece was rated by 182 participants and at least six pieces (= 50 %), were rated by 160 participants.

1.1.3 Sample Characteristics

Table 1 gives information about the musical skills of the musicians' groups. While groups differed in their musical training, no difference was found for the self-reported perceptual abilities.

	Professional jazz musician ($N = 39$)		Semiprofessional jazz musician ($N = 24$)		Amateur jazz musician ($N = 47$)		Non-jazz musician ($N = 42$)		jazz-loving non-musician ($N = 8$)	
	M	*SD*	*M*	*SD*	*M*	*SD*	*M*	*SD*	*M*	*SD*
Hours of daily practice	2.9	2.6	2.8	5.9	0.9	0.8	1.0	1.0	0.0	0.0
Concerts played (last 12 months)	55.4	46.9	32.1	33.6	7.1	8.4	12.1	18.6	0.0	0.0
Daily regular practice (in years)	-	-	-	-	14.0	11.9	12.5	9.1	1.3	2.3
Number of instruments	-	-	-	-	2.9	1.6	3.3	1.5	0.6	1.1
Daily* practice of primary instrument (in h)	-	-	-	-	2.0	1.5	2.5	1.4	0.9	1.0
Formal training in music theory (in years)	-	-	-	-	2.9	4.6	3.1	4.2	0.4	1.1
Formal training on instrument (in years)	-	-	-	-	8.4	5.8	9.6	6.0	2.1	4.8
Perceptual abilities (mean score)	-	-	-	-	4.5	0.8	4.5	0.5	4.5	1.1

Note. * at the peak of their interest.

Table 1: Musical Background of the sample. After self-classification into musicians' groups, participants provided further background about their musical training. Professional and semiprofessional jazz musicians stated their current daily practice and the number of concerts they played within the last year. All other groups additionally filled out items taken from two sub-scales of the Gold-MSI (musical training and perceptual abilities) to provide further details about their skill levels. The items of the musical training scale used are individually presented in the table. For the perceptual abilities, the mean (min = 1, max = 7) of the nine items was calculated.

1.2 Study Design

In the listening study, we used a within-between subject mixed design: By multiplying the twelve recordings by four different versions (the original version and the three manipulations quantized, exaggerated and inverted), we obtain 48 stimuli in total. The stimuli were divided into four stimulus sets, to which participants were randomly assigned. Every set contained four pieces in the original version, four in the quantized, four in the exaggerated and four in the inverted version. Therefore, every set contained all manipulations and pieces but never two versions of the same piece (See Figure 1). Each set was divided into two blocks of six pieces. This was done to prevent that the same versions would be presented subsequently. Also, this enabled the analysis of participants who dropped out but evaluated at least half of the pieces. The order of blocks and the order of pieces within blocks were randomized. Every piece was presented and evaluated individually.

1.3 Descriptive Statistics: Distribution of the Swing Ratings

Figure 2 shows the proportion of the participants' ratings across pieces for each point on the swing scale. Exaggerated versions were rated the least and quantized version the most swinging. Original recordings and their inverted versions have similar distributions and were both rated as more swinging compared to the exaggerated but not as much as the quantized versions. Additionally, a difference between the musicians' groups was found. Professional jazz musicians gave overall lower swing ratings, independently of the version of a piece. Randomization of stimuli-sets caused that in the group of jazz-loving non-musicians, which consisted only of eight participants, not all versions of a piece were evaluated. This group was excluded from the inferential statistical analysis.

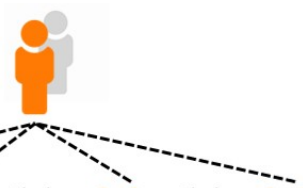

Piece	Tempo	Set 1	Block	Set 2	Block	Set 3	Block	Set 4	Block
Doxy	130	original	1	quantized	1	exaggerated	2	inverted	1
Alfie's Theme	135	quantized	2	exaggerated	1	inverted	1	original	2
Paper Moon	135	exaggerated	2	inverted	2	original	1	quantized	1
Blue Monk	140	inverted	1	original	2	quantized	2	exaggerated	2
Serenade to a Cuckoo	140	original	2	quantized	2	exaggerated	1	inverted	1
Don't Get Around*	140	quantized	1	exaggerated	1	inverted	2	original	2
Jordu	150	exaggerated	2	inverted	2	original	1	quantized	1
So What	160	inverted	1	original	1	quantized	2	exaggerated	2
In a Mellow Tone	160	original	1	quantized	1	exaggerated	1	inverted	2
Four	170	quantized	2	exaggerated	2	inverted	2	original	1
Yardbird Suite	180	exaggerated	1	inverted	1	original	2	quantized	2
Now's The Time	190	inverted	2	original	2	quantized	1	exaggerated	1

Figure 1: Study Design. Participants were randomly assigned to one of four stimulus sets. Every stimulus set contained all 12 pieces, but in a different version. Sets were divided into two blocks, each containing six pieces. The order of blocks and pieces within a block was randomized for each participant. * The piece *Don't get around much anymore* was shortened to fit.

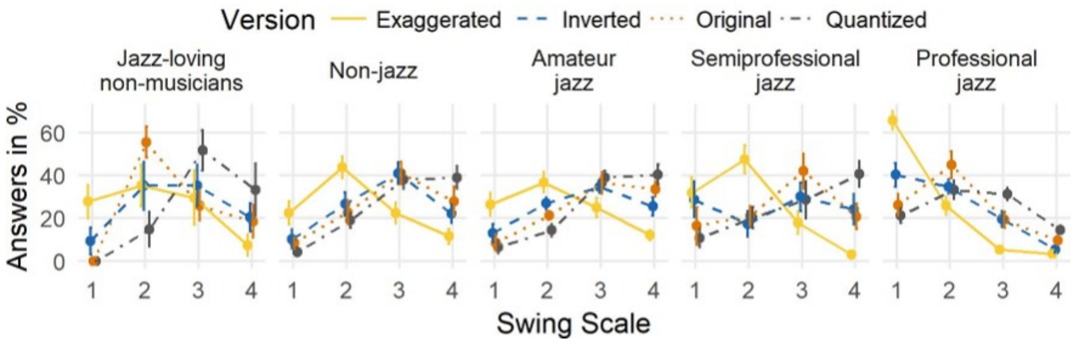

Figure 2: Distribution of the swing ratings across all musicians' groups including jazz-loving non-musicians. On every point of the four point scale for *swing* (from 1 = not at all to 4 = very much) the percentage of participants, who chose this option, is shown. Error bars reflect standard errors of the mean (between-pieces).

1.4 Cumulative Link Mixed Model

The cumulative link mixed model was fitted using the *clmm* function from the ordinal package in *R* [1]. Likelihood-ratio tests (LRT) were used for the model selection. LRT compared the likelihood of a full model with three fixed effects condition (original, quantized, exaggerated, inverted), musicians' category and their interaction against partial and no-covariate models in which one of the predictors was removed. Parameter estimates of the full model are presented for naturalness in Table 2 and for technical correctness in Table 3.

1.5 Receiver Operating Characteristics (ROC) Analysis

1.5.1 Swing

Response data of the swing ratings were analyzed by ROC analysis, results of which indicated the discriminability between original versions of individual pieces and their different manipulations. For each music piece, pairs of the relative frequencies of each response criterion on the swing scale (from 1, "not at all" to 4, "very much") were generated for the original version of a piece and the manipulations. Additionally, the averages for the comparison across pieces were calculated. These pairs were then plotted to form ROC curves, presented in Figure 7 for the individual pieces: The discriminability between manipulation and the original version are quantified by the area under the ROC curve (AUC; specified in each individual graph). Measures below 0.5 reflect higher swing ratings for the original version and measures above 0.5 higher swing ratings for the manipulations, respectively. For estimating the 95% confidence intervals the DeLong method [2] was used. Aggregating over pieces, quantized versions elicit higher swing ratings compared to the original versions (AUC= .57, CI= .53-.60). The opposite is found for the

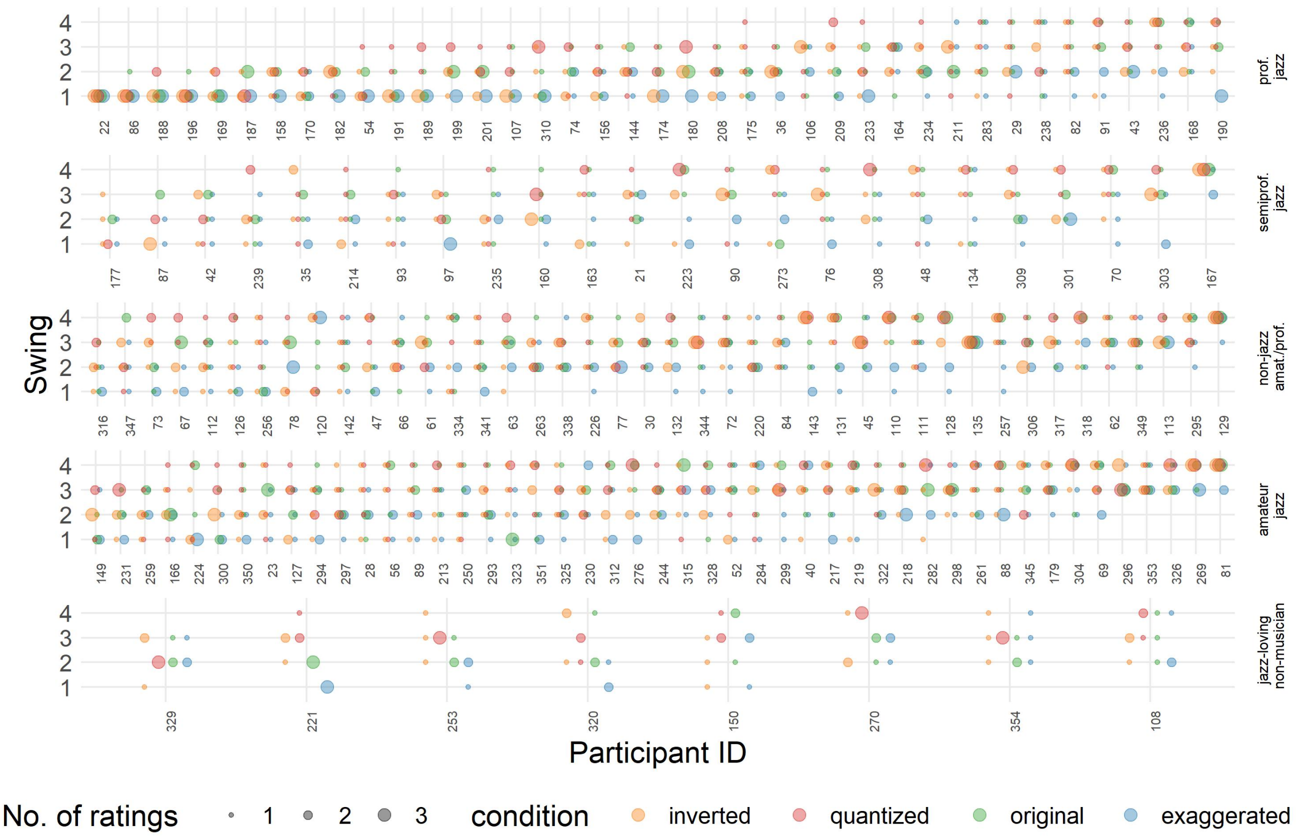

Figure 3: Consistency of swing ratings (single subject level). Swing ratings for each condition are shown separately for every participant. The rating scale is from 1 (not at all swinging) to 4 (very much swinging). Participants are grouped by their musicians' category. Every participant rated three pieces per condition, resulting in three observations per condition. In this graph, the sizes of the points indicate the number of pieces with the same rating, i.e. the largest point shows that all three pieces in this condition were rated the same by the respective participant. On the x-axis, the participant-ID is sorted by the participant's overall swing ratings. Participants giving rather low swing ratings (independently of the condition) are at the lower end, participants giving higher swing ratings on the upper end of the axis. Please note that the jitter on the x-axis is only for better discriminability between conditions.

	β	*SE*	p (β)	χ^2 (df)	p (χ^2)	Nagelkerke's pseudo R^2
Condition				134.03 (3)	< .001	.078
exaggerated	-1.306	0.238	<.001			
inverted	-0.444	0.236	.060			
quantized	-0.020	0.240	.935			
Musicians' Category				46.76 (3)	<.001	.104
amateur jazz	0.212	0.387	.575			
professional jazz	-2.064	0.389	<.001			
semiprof. jazz	-0.452	0.455	.320			
Condition x Musicians' Category				5.85 (9)	.756	.107
Threshold coefficients	Estimate	*SE*				
1|2	-3.239	0.308				
2|3	-1.186	0.298				
3|4	0.839	0.297				

Table 2: Proportional odds mixed model for naturalness. Analogously to the swing criterion, three models were tested with Likelihood-Ratio-Tests (LRT). The first model considered the condition only, the second both, condition and musician's group. In the third model the interaction term for manipulation and musician group was tested as well. Estimates (β) and standard errors are shown for the complete model. As references, the original version of a piece and the group non-jazz musicians were used.

	β	SE	p (β)	χ^2 (df)	p (χ^2)	Nagelkerke's pseudo R^2
Condition				537.62 (3)	< .001	.283
exaggerated	-2.532	0.262	<.001			
inverted	-0.703	0.254	.001			
quantized	0.241	0.258	.351			
Musicians' Category				25.27 (3)	<.001	.294
amateur jazz	0.207	0.400	.605			
professional jazz	-1.297	0.415	.002			
semiprof. jazz	-0.088	0.479	.854			
Condition x Musicians' Category				8.76 (9)	.460	.298
Threshold coefficients	Estimate	*SE*				
1|2	-4.759	0.341				
2|3	-2.179	0.319				
3|4	0.351	0.314				

Table 3: Proportional odds mixed model for technical correctness. For the question, whether pieces were perceived as technically correctly played, three models were tested with Likelihood-Ratio-Tests (LRT). The first model tested condition only, the second both, condition and musician's group. In the third model the interaction term for manipulation and musician group was included. Estimates (β) and standard errors are shown for the complete model. As references, the original version of a piece and the group non-jazz musicians were used.

exaggerated versions (*AUC*= .30, *CI*= .27-.34). Overall, inverted versions were rated less swinging compared to the original (*AUC*= .45, *CI*= .41-.48). However, there is only a slight tendency towards the preference of the original versions when excluding the two pieces *Jordu*, and *Yardbird Suite* (*AUC*= .47, *CI*= .43-.51). Across pieces, a variation in the discriminability between versions was observed. Overall, AUC for the individual pieces ranged from 0.50 (*Don't get around much anymore*) to 0.65 (*Now's the time*) for quantized vs. original, from 0.18 (*Alfie's Theme*) to 0.42 (*Now's the time*) for exaggerated vs. original and from 0.32 (*Jordu*) to 0.56 (*Four*) for inverted vs. original versions.

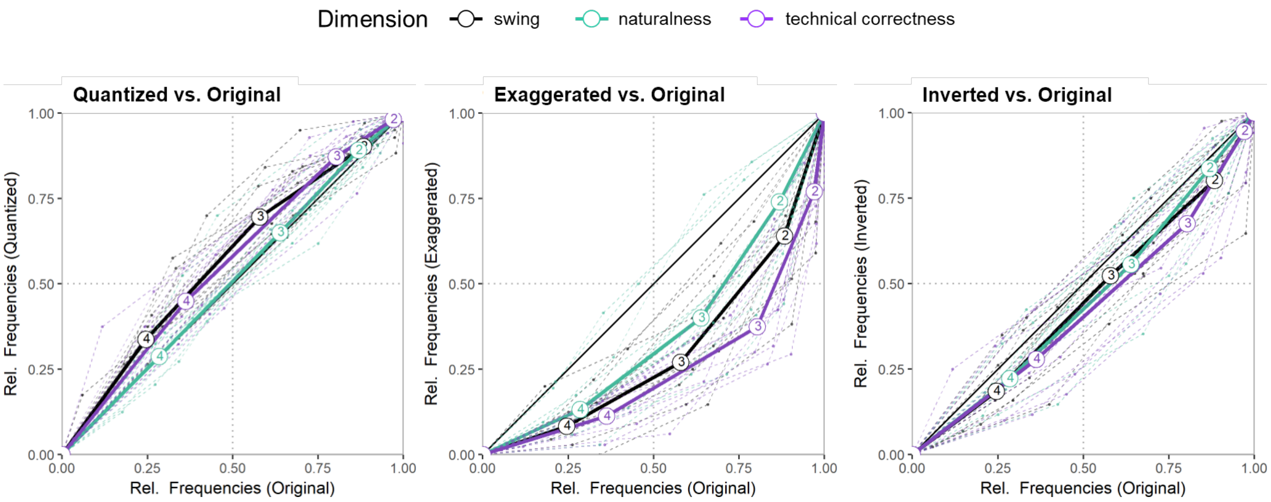

Figure 4: Receiver Operating Characteristic (ROC) curves for all three dimensions (swing, naturalness and technical correctness) across pieces.

1.5.2 All Dimensions: Swing, Naturalness and Technical Correctness

To better understand the swing ratings, we implemented two control questions in our survey. For the case that quantized versions would swing less than the original pieces, we expected the quantized to score high on technical correctness (because there were no timing errors at all) but lower on naturalness. If there were differences between inverted and original versions, we would have expected to find lower swing ratings and lower ratings on naturalness for the inverted compared to the original versions. The question about technical correctness was mainly implemented to control for perceived errors of the pianist's playing in the original pieces. Although we tried our best to select

	Comparison	AUC_{swing}	AUC_{nat}	AUC_{tech}	z	p
original vs. quantized	swing vs. nat	0.57	0.51	-	4.08	$< .001$
	swing vs. tech	0.57	-	0.56	0.61	1.000
	nat vs. tech	-	0.51	0.56	-2.59	.085
original vs. exaggerated	swing vs. nat	0.30	0.36	-	-3.68	.002
	swing vs. tech	0.30	-	0.25	3.17	.014
	nat vs. tech	-	0.36	0.25	6.02	$< .001$
original vs. inverted	swing vs. nat	0.45	0.45	-	-0.21	1.000
	swing vs. tech	0.45	-	0.43	1.21	1.000
	nat vs. tech	-	0.45	0.43	1.35	1.000

Table 4: DeLong Tests for ROC curve comparisons. Estimates for the pairwise DeLong tests for correlated ROC curves are displayed for each comparison between dimension (swing, naturalness and technical correctness) and the comparisons for manipulations and original versions. P-values are Bonferroni adjusted. AUC = Area under the curve.

parts of the recording where none of such errors occurred, irregularities in single bars or phrases might be perceived as errors by some of the participants. We hoped to be able to better discriminate, whether low swing ratings were caused by local errors or something else. To not give away information about the manipulation, we had to always ask all questions. The data shows that the three dimensions are highly correlated (swing and naturalness, $r = .66$; swing and technical correctness, $r = .51$; naturalness and technical correctness, $r = .45$). Figure 4 displays ROC curves for all three dimensions (swing, naturalness and technical correctness) across all pieces for the comparisons of the manipulation and original versions. We performed pairwise comparisons of DeLong [2] tests for correlated ROC curves, which test whether the true difference in the area under the curves (AUC) is equal to 0 (see Table 4). The results suggest, that for the discrimination between original versions and quantized versions, swing and naturalness are not identical. Whereas swing differentiates between versions of a piece, naturalness does not, similarly to the exaggerated versions. Here, swing also discriminates better between manipulations compared to naturalness. Additionally we find for exaggerated versions, that the question about technical correctness differentiates even stronger than swing. No additional information of the two control questions can be derived for the inverted versions.

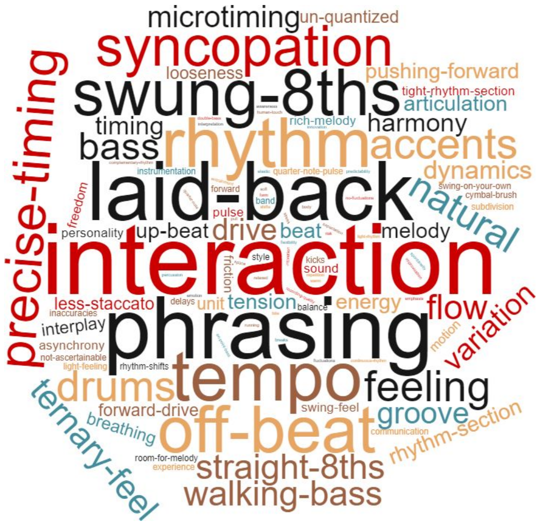

Figure 5: Word cloud. The participants' answers to the question "What do you think makes a piece of music swing?" were first summarized, categorized and subsequently put into a word cloud. More frequently mentioned terms are displayed larger.

1.6 Exploratory Model Selection to Predict Swing

In an exploratory approach, properties of MTDs were included in a model to predict swing. In the first step we pre-selected theoretically relevant variables, as there were the standard deviation of the average swing ratio, the standard deviation of base notes and swing notes in milliseconds, and the mean deviation of the MTDs of the base notes and those of the swing notes, both in ticks. In the second step, the best predictors and their sequence were then automatically selected in a data-driven approach. The computation was automated with the R package MuMIn[3],

which in this case sorts models according to Akaike's Information Criterion (AIC,[4]). Selecting models based on data has several limitations (e.g. risk of over-specification) and interpretations should only made cautiously. Table 5 shows the parameters of the estimates for the best model, which included only three predictors in this order: a) Mean MTDs of the base notes, b) standard deviation of MTDs of base notes and c) mean MTDs of the swing note. Only mean MTDs of base note was positively related to swing ($OR = 2.42$, $CI = 1.69$ - 3.45, $p < .001$). The standard deviation of MTDs of base notes ($OR = 0.58$, $CI = 0.52$ - 0.65, $p < .001$) and the mean MTDs of swing notes ($OR = 0.64$, $CI = 0.48$ - 0.84, $p = .002$) were both negatively related to swing. Since we have not systematically manipulated these parameters, no generalizing conclusions can be drawn from the results. Nevertheless, it would be interesting to further investigate the delay of base notes, which might be related to a laid-back feel, with a larger number of samples. Also the negative impact of the delay of swing notes and the variability of the base note MTDs should be explored in more detail. Interestingly, the variation of the swing ratio was not selected among predictors. One possibility for this finding is, that we did not measure the local stability of the swing ratio, which might be the better indicator than the standard deviation of the swing ratio.

	β	SE	p (β)	χ^2 (df)	p (χ^2)	Nagelkerke's pseudo R^2
b_mtd_mean_t	0.884	0.182	$< .001$	181.92 (1)	$< .001$	.104
b_mtd_std_ms	-0.541	0.059	$< .001$	99.02 (1)	$< .001$	.156
s_mtd_mean_t	-0.453	0.142	.001	10.27 (1)	.001	.161

Table 5: Proportional odds mixed model with MTDs characteristics. Model parameters of the best model resulting from an automated model selection. Individual predictors were subsequently tested with Likelihood-Ratio-Tests (LRT). The first model considered the first predictor only, the second both and the third model all three predictors. Estimates and standard errors are shown. b_mtd_mean_t = mean MTDs of base notes in ticks, b_mtd_std_ms = standard deviation of MTDs of base notes in milliseconds, s_mtd_mean_t = mean of MTDs of swing notes in ticks.

1.7 Visual inspection of proportional odds assumptions

See Figure 6.

1.8 Participants' Comments

At the end of the online survey, participants could write what in their point of view makes a piece of music swing. This question was non-obligatory, yet 106 participants shared their opinion with us. We attempted to categorize and summarize the answers, which were then "quantified" in a Word cloud (see Figure 5). The more frequently a term/concept is mentioned, the larger the word is displayed in the Figure. The aim was not to create a complete classification, but to highlight the aspects that the musicians consider particularly important. Only few participants stated that swing is hard to grasp, define or that it "can't be taught" or "learned". There were many comments about what swing entails: that it is a kind of feeling, groove and flow. Many participants additionally provided detailed descriptions of components and possible mechanisms for swing. Several not rhythm-related aspects were mentioned, from instrumentation, sound and the quality of recordings, harmonization and variation in the melody to the personality of musicians. Yet, most of the answers were about rhythm. Important components of swing appear to be phrasing and the interaction between musicians, between the rhythm section and the soloists and within the rhythm section. However, we do not see full agreement on how this communication and interaction should be shaped exactly. Precise timing, consistency and no fluctuations are contrasted against microtiming, looseness and "un-quantized" tracks. For one person, even inaccuracies were considered necessary for swing. Many participants put much effort in their answers and described more precisely how asynchrony between the musicians should be designed to create a laid-back-feel or forward-drive. It was mentioned several times that asynchrony creates room for the melody and allows the music to breathe. Syncopation, rhythmic variation and alternation between straight-8th notes and swung-8th notes were frequently mentioned. Typical elements for swing like a walking bass, the importance of off-beats or a (at least) moderate tempo were also discussed. The original comments and a translation can be downloaded via the following link ().

2 Recording and Manipulating

A unique professional bass and drum track was written for each piece, using human performances. Both were quantized to 8th note triplets. The pianist was listening to these two tracks while being recorded, effectively playing with a metronome. In addition, the pianist had as much freedom as possible for the performance. The only restraint in the recordings was that ornaments (like e.g. trills, acciaccaturas, etc.) were to be avoided, as they don't have a

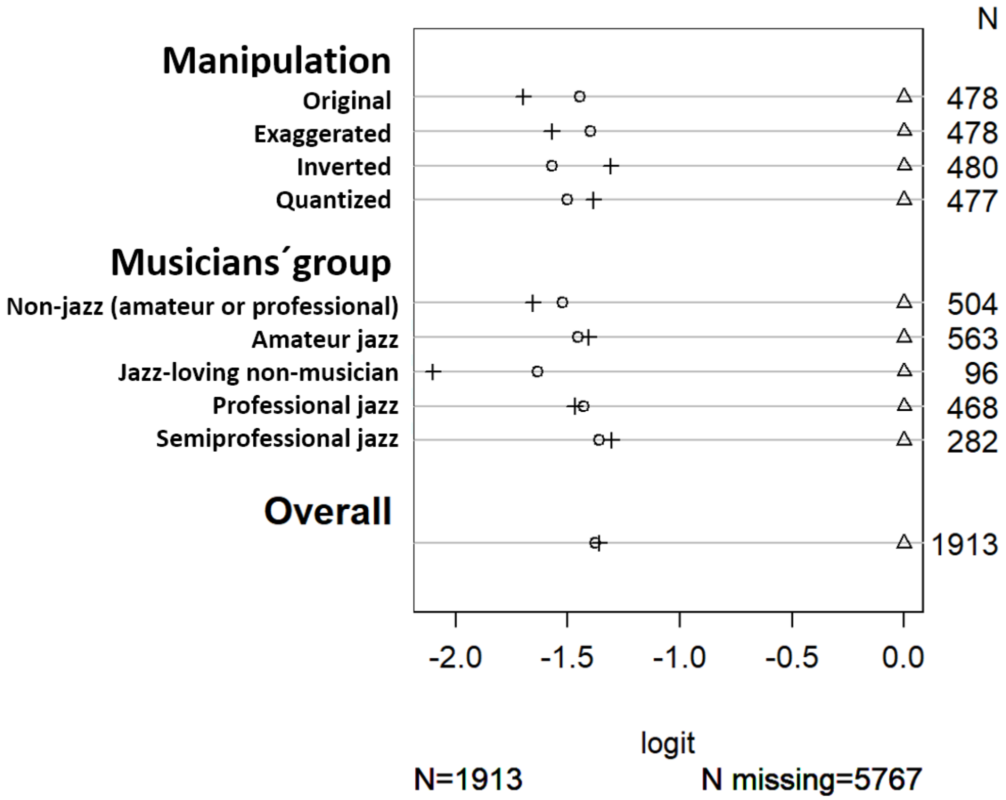

Figure 6: Equality of coefficients across cutpoints of the ordinal regression. For the predictor variables condition (original, quantized, exaggerated and inverted) and musicians' group, distances between the symbols for each set of categories of the dependent variable, should remain similar, if the proportional odds assumption holds. The first set of coefficients was normalized to be zero (common reference point for better visibility). The distance between the sets of coefficients for the variable condition is similar. In contrast, the distances between the estimate for the group "jazz-loving non-musicians) are different from the other musicians' group, suggesting that the proportional odds assumption may not hold.

well defined temporal position. It should be noted that the pianist *could* play full 8th note triplets, but he chose to play mostly swung 8th notes. Finally, we did not distinguish between notes played with the left hand (chords) or the right hand (melody), so they are all considered belonging to the same series of notes. For each recording the recorded MIDI (Musical Instrument Digital Interface) piano track was exported and then manipulated as described in the main text. It was then re-imported into our recording software (in this study Cubase 8.5 Pro) to produce audio files. For transforming MIDI to audio we used the following virtual instruments: Addictive Drums 2, Addictive Keys, BASiS.

3 Detailed definition of Microtiming Deviations and the tick unit

Since the recordings are in the form of MIDI data, we measure the temporal position of notes in *ticks*. This is a dimensionless unit of measuring time, native to music, which the MIDI standard uses. Each tick is equal to 1/960-th of a quarter note and can be transformed to milliseconds once the beats per minute (BPM) are given since 1 tick = (62.5/BPM) ms. A MIDI note's position is given in ticks with respect to 0, with 0 ticks meaning the beginning of the song. A duration of a MIDI note is also given in ticks, however the note duration was not manipulated in our study.

As mentioned in the main text, we define MTDs individually for the "base" notes b and "swing" notes s. Thus, as a first step, we classify note events based on their position in ticks: Let p_i be the true position of a note (in ticks) and

$$f(p_i) = \begin{cases} (p_i \bmod 960) - 960 & \text{if } (p_i \bmod 960) \geq 800 \\ (p_i \bmod 960) & \text{else} \end{cases} \tag{1}$$

where $(a \bmod b)$ is the remainder after dividing a by b. f brings all notes into a single quarter note and its action is shown in Fig.1**B** of the main text. Notes are then categorized according to which part of the 8th note triplet they fall into: the *set* of "base" notes b has $f(b) \in [-160, 160)$, while the *set* of "swing" notes s has $f(s) \in [480, 800)$, see Fig. **??B**.

In the second step, we calculate (for each note type) the mean note position as

$$\bar{b} = \frac{1}{||b||}\sum_{i\in b} f(p_i)\,, \quad \bar{s} = \frac{1}{||s||}\sum_{i\in s} f(p_i) \tag{2}$$

with $||\cdot||$ the cardinality of a set. The MTDs of note i is $d_i = f(p_i) - \bar{b}$ or $d_i = f(p_i) - \bar{s}$ depending on note category. Notes with $f(p_i) \in [160, 480)$ (i.e. they fall in the 2nd part of the triplet) are so few that they are disregarded. To compute σ in Table **??** we translate MTDs to milliseconds by multiplying them with (62.5/BPM) for each piece.

4 Possible correlations of the AUC with other measures

In this section we compare the Area Under Curve (AUC) defined in the preceding sections, with various measures that can quantify musical aspects of a recording. We chose to compare the AUC of the quantized versus original version. The results are shown in Fig. 8. This comparison was mainly done as a “sanity check”, to ensure that there is no other reason that the quantized versions are preferred (besides the obvious absence of microtiming deviations).

The measures used are as follows:

1. BPM: beats per minute (recording tempo).
2. r: average swing ratio, as defined in main text.
3. δ_r: std. deviation of r.
4. n.p.q.: Average number of notes played in one quarter note. This is a measure of the complexity of the piece.
5. $||s||/||b||$: ratio of total amount of swing notes towards total amount of base notes.
6. $\bar{v}$: average velocity of notes. “Velocity” is a MIDI term that corresponds to with how much intensity is each note played.
7. δ_v: std. of note velocity.
8. k_v: skewness of the note velocity distribution.
9. $\bar{b}$: mean position of base notes, as defined in main text.
10. σ_b, σ_s: std. of MTDs of base/swing notes respectively, as defined in main text.
11. r_2: a second measure for the average swing ratio. It is simply the swing ratio of $\bar{b}$ and $\bar{s}$.

References

[1] R. H. B. Christensen. ordinal—regression models for ordinal data, 2018. R package version 2018.4-19. http://www.cran.r-project.org/package=ordinal/.

[2] Elizabeth R DeLong, David M DeLong, and Daniel L Clarke-Pearson. Comparing the areas under two or more correlated receiver operating characteristic curves: a nonparametric approach. *Biometrics*, pages 837–845, 1988.

[3] Barton K. Mumin, multi-model inference. r package version 1.15. 6.; 2016, 2016.

[4] Hirotogu Akaike. Information theory and an extension of the maximum likelihood principle. In *Breakthroughs in statistics*, pages 610–624. Springer, 1992.

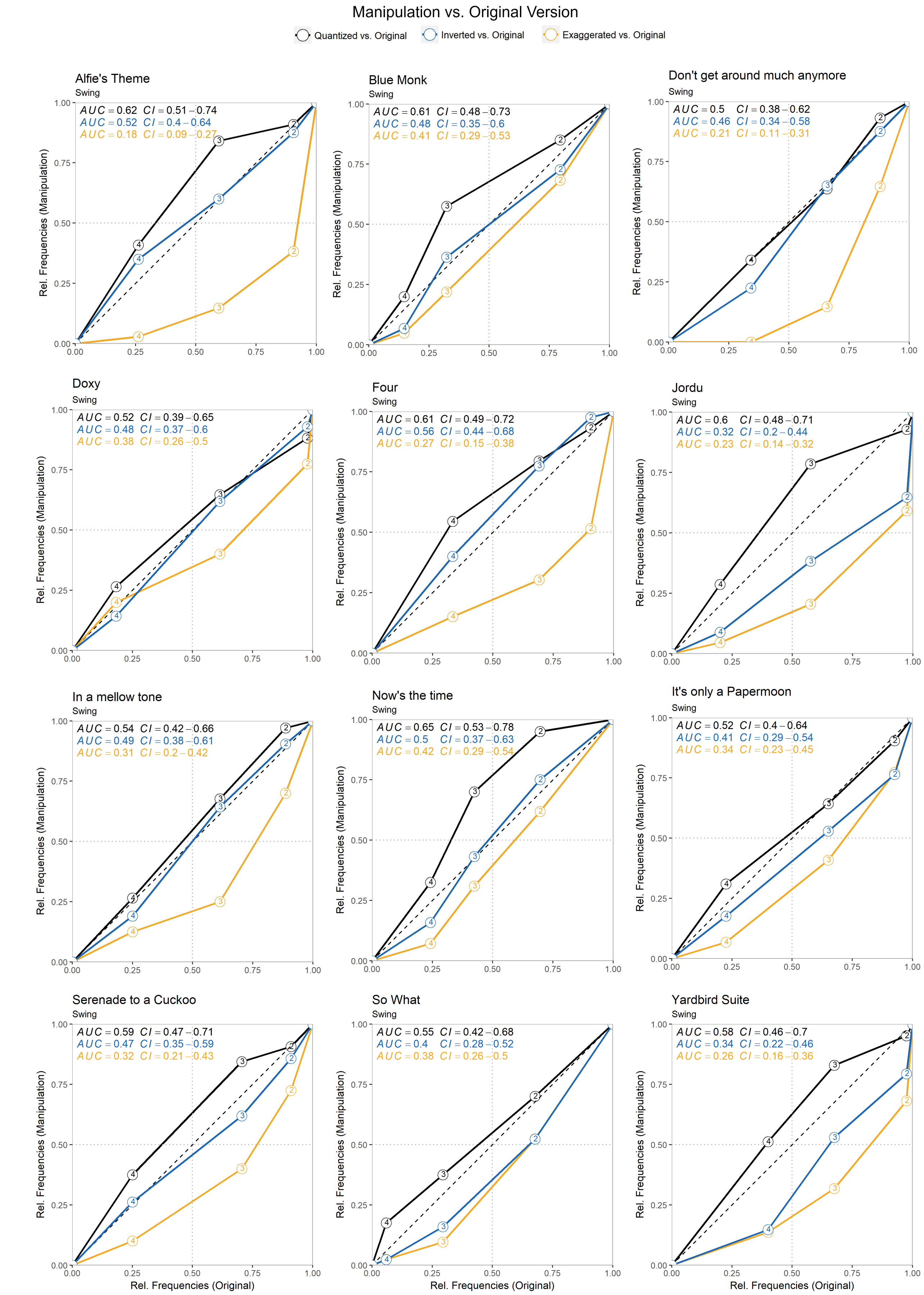

Figure 7: Receiver Operating Characteristic (ROC) curves for swing. For each piece and condition, ROC curves were drawn. The discriminability between original versions and the respective manipulation are indicated by the area under the curve (AUC). The curves display cumulative proportions for each answer category, from 4 (very much) to 1 (not at all), of which the manipulation is plotted on the original version. AUC below 0.5 indicate a preference for the original version.

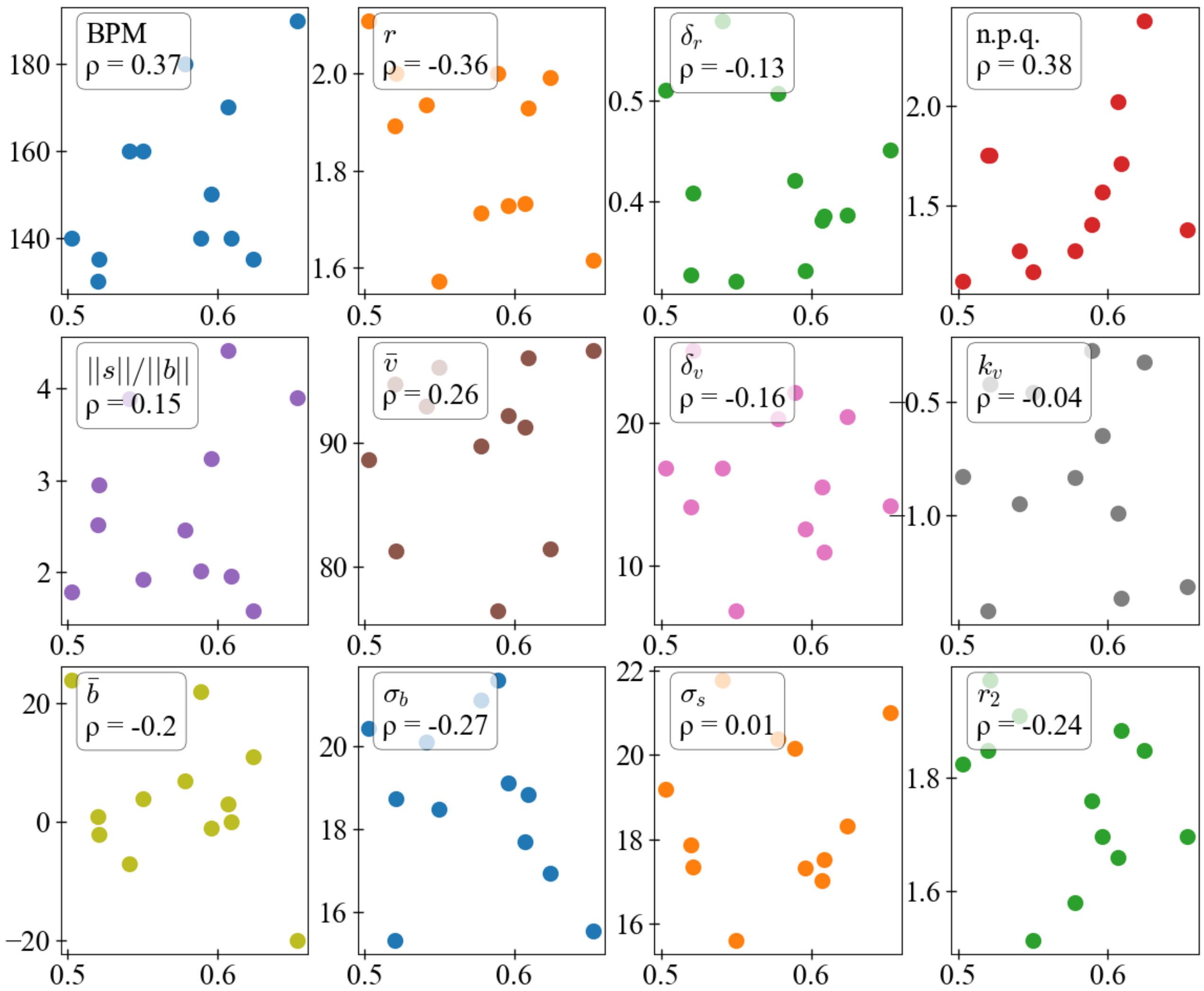

Figure 8: Scatter plots of various measures quantifying a recording, versus the AUC of quantized-original versions. Each dot represents a recording. The plot labels also show the Spearman's rank correlation coefficient ρ. For details of each measure please see section 4.
